# Supplementary material for: A polyvalent inactivated rhinovirus vaccine is broadly immunogenic in rhesus macaques
Source: Nat Commun. 2016 Sep 22;7:12838. doi: 10.1038/ncomms12838 (PMC5036149; doi:10.1038/ncomms12838)
Supplement: Supplementary Information — Supplementary Figures 1-7, Supplementary Tables 1-4 and Supplementary References [file ncomms12838-s1.pdf]

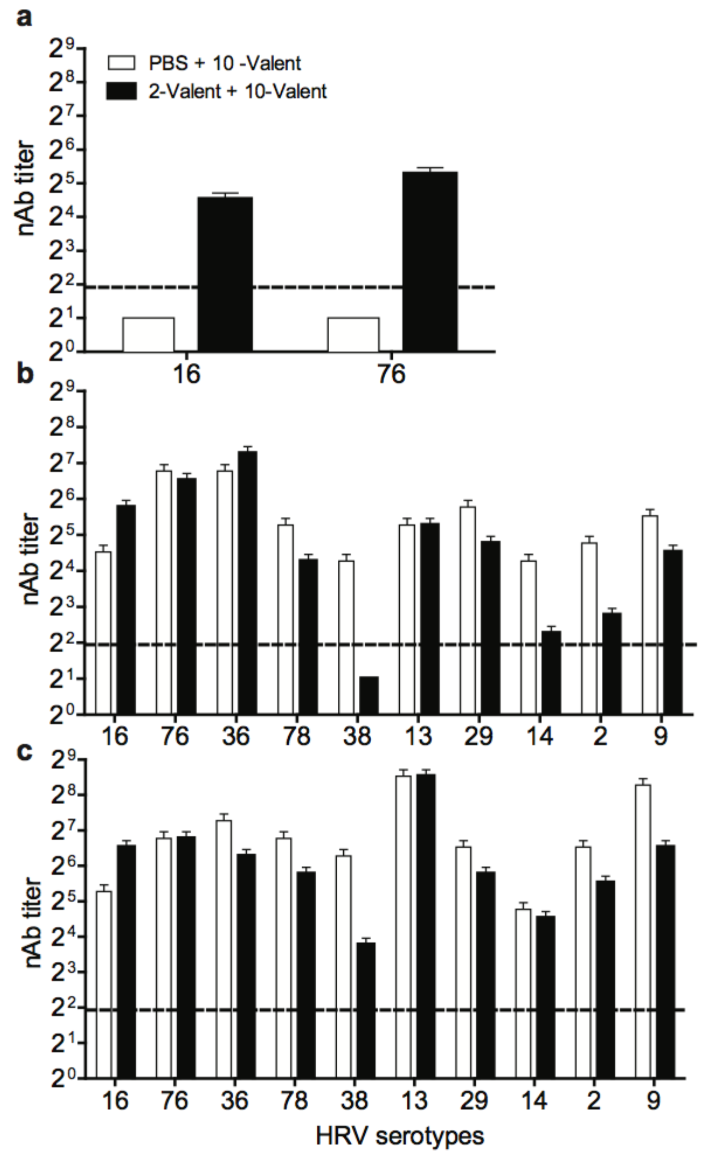

Supplementary Fig. 1. Original antigenic sin in 10-valent inactivated HRV-vaccinated mice. Mice (20 per group) were vaccinated i.m. with either PBS + alum or 2-valent (HRV-16 and HRV-76) inactivated HRV + alum in order to establish pre-existing immunity to types 16 and 76 in one group. (a) Sera were collected 18 days later and tested for nAb against HRV-16 and HRV-76. All mice were vaccinated i.m at day 28 and boosted at day 56 with 10-valent inactivated HRV that included types 16 and 76 (10-valent composition listed in **Supplementary Table 2**). Sera were collected at days 46 (b) and 74 (c) and pooled for each group for nAb against the 10 types in the vacccine. Error bars show 95% confidence interval. The dashed line represents LOD. Undetectable nAb were assigned LOD/2.

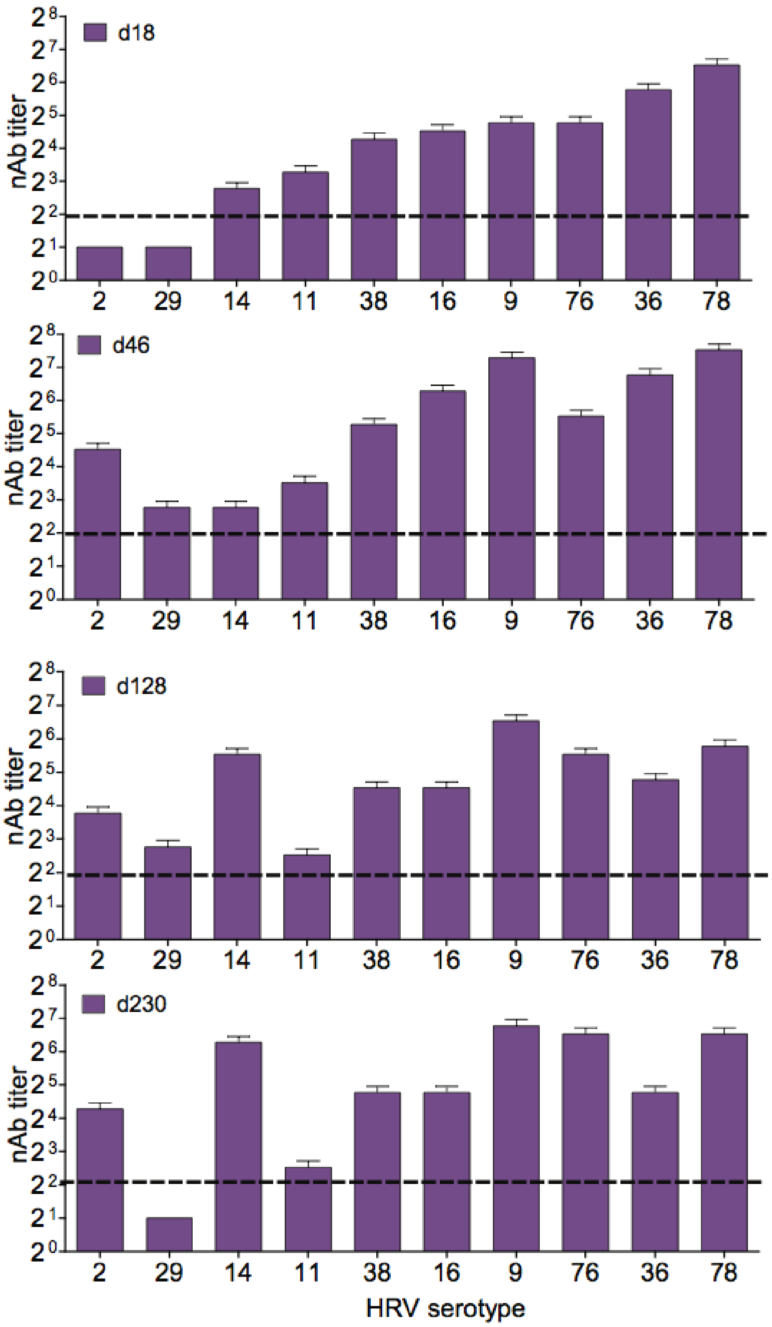

Supplementary Fig. 2. Durability of nAb response to 10-valent inactivated HRV. 20 mice were vaccinated then boosted at day 28 with 10-valent HRV. The inactivated-$TCID_{50}$ input titers per dose are specified in **Supplementary Table 2**. Sera were collected at days 18, 46, 128, and 230, and nAb titers against the HRV types in the vaccine were determined in pooled sera. Error bars depict 95% confidence interval. The dashed line represents LOD. Undetectable nAb were assigned LOD/2

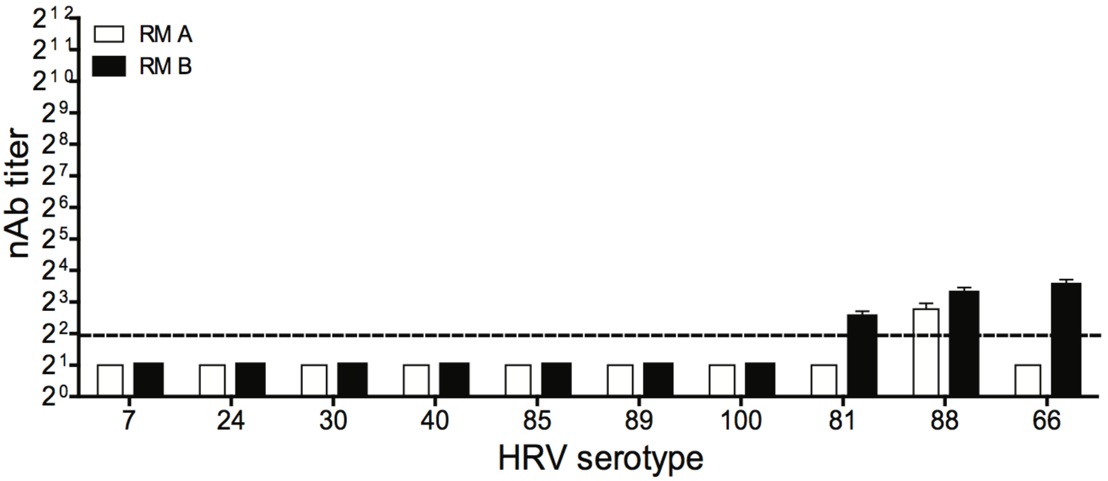

Supplementary Fig. 3. Minimal cross-neutralizing antibodies in rhesus macaques anti-sera. Two rhesus macaques (RM A and RM B) were vaccinated i.m. with 25-valent HRV + alum. The RM received an identical boost vaccination at day 28, and sera were collected at day 46. Serum nAb titers were measured against 10 types not present in this vaccine (**Supplementary Table 3**). Error bars depict 95% confidence interval. The dashed line represents LOD. Undetectable nAb were assigned LOD/2.

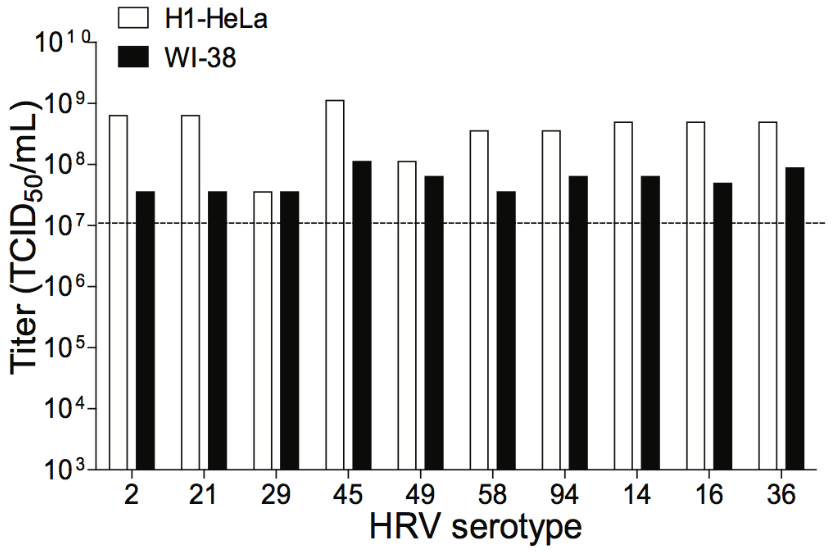

Supplementary Fig. 4. Infectious yield of HRV types in WI-38 cells. Ten representative types were chosen from the 25-valent vaccine composition (**Supplementary Table 2**). Infectious titers of the indicated HRV types in stocks produced from HeLa and WI-38 cells infected at MOI = 0.1. The grey line at 10^7 $TCID_{50}$/ml shows the target titer. The experiment was replicated, and similar results were observed.

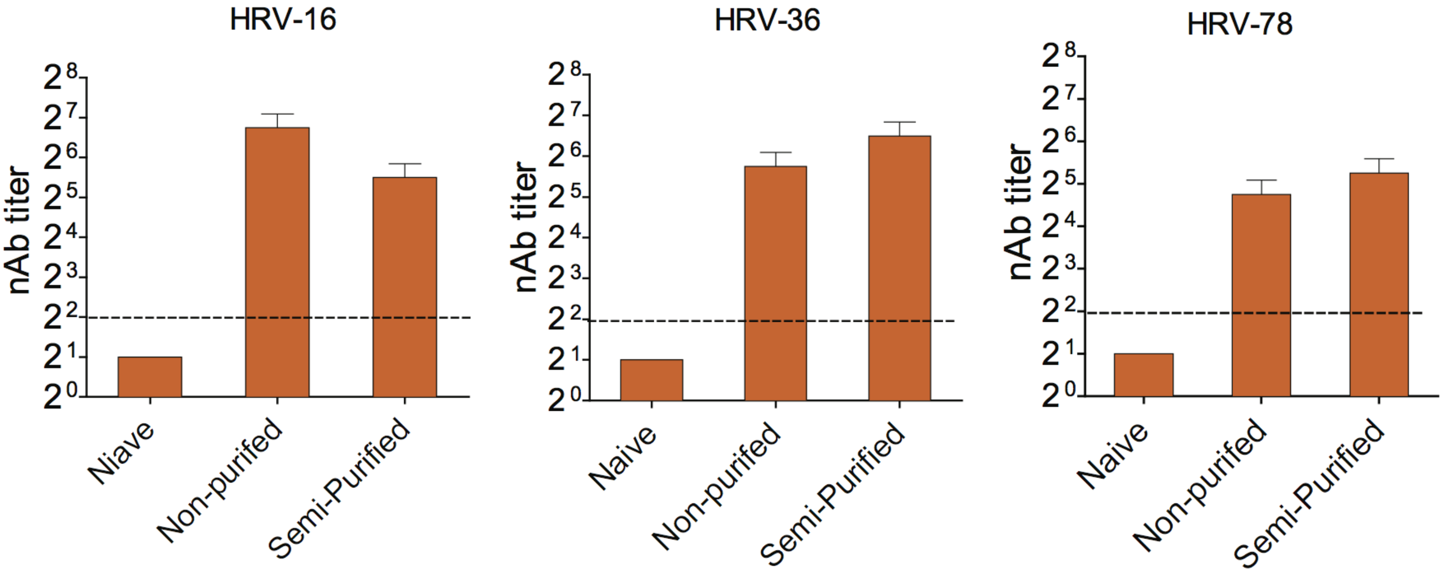

Supplementary Fig. 5. Comparison of immunogenicity of inactivated semi-purified HRV and inactivated non-purified HRV. HRV-16, HRV-36, and HRV-78 virus stocks were semi-purified as described in the Methods. Mice were vaccinated i.m. with either semi-purified 3-valent inactivated HRV with alum (20 mice) or non-purified 3-valent inactivated HRV with alum (20 mice). HRV types and inactivated-TCID50 doses are specified in **Supplementary Table 4**. Sera were collected 18 days after vaccination and pooled for each group. Serum nAb titers were measured against HRV-16, HRV-36, and HRV-78. As a negative control, sera from naïve mice were used. Error bars show 95% confidence interval. The dashed line represents LOD.

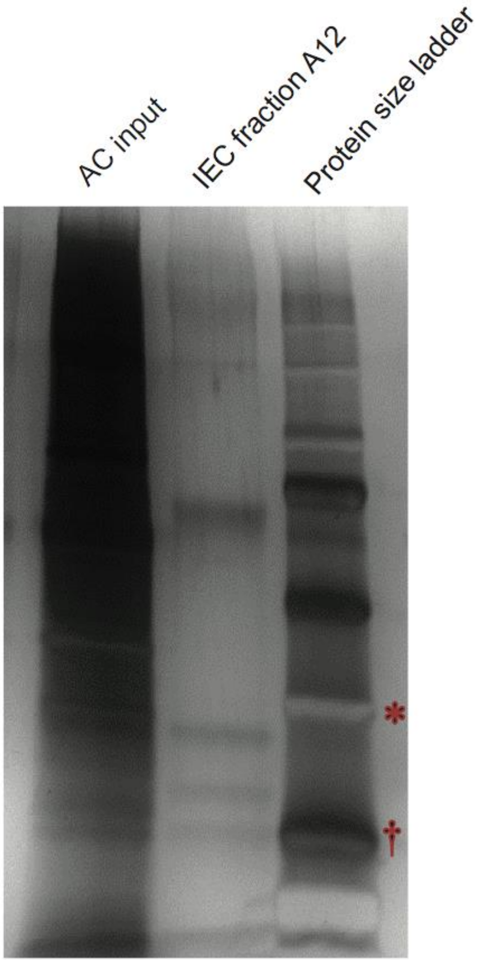

Supplementary Figure 6. Purity of HRV-36 after high performance liquid chromatrography. HRV-36 propagated in H1-HeLa cells (see methods) was loaded through a HiTrap Blue HP affinity chromatography column (AC) to remove serum albumin. The input was 15 ml and 6.3 × 10^8 $TCID_{50}$/ml. The virus-containing AC flowthrough was collected and loaded through a HiTrap Capto Core 700 size exclusion column (SEC). The SEC flowthrough was dialyzed overnight at 4°C in 0.1M Tris-HCl pH8 buffer, and loaded the next day on a HiTrap Q XL ion exchange column (IEC). Fractions were eluted with a 0.1M Tris-HCl pH8 buffer on a NaCl gradient; fractions showing high absorbance at 280 nm were collected for analysis. Purity of chromatographic fractions was visualized by silver stain on a 10% SDS-PAGE gel. In the representative gel, IEC fraction A12 showed reduced cell protein compared to input virus stock (AC input) and distinct bands corresponding to predicted sizes of HRV-36 capsid proteins[3, 4]. Fraction A12 was 2 ml and 1.1 × 10^9 $TCID_{50}$/ml. *, 37 kDa. †, 25 kDa.

a

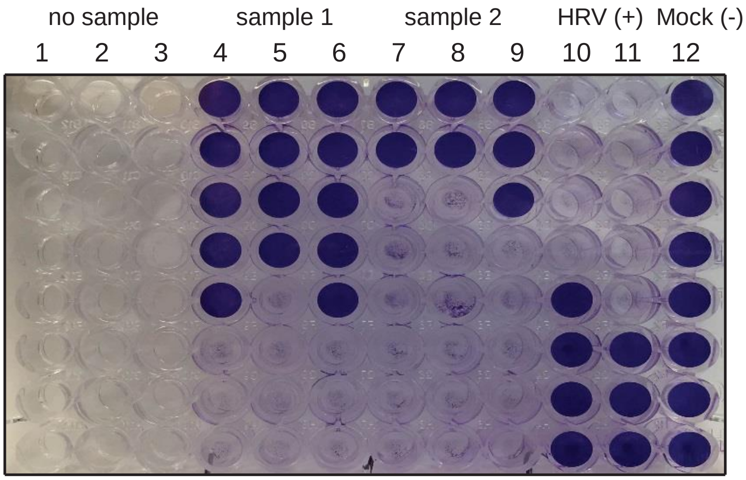

b

	no sample			sample 1			sample 2			HRV (+)		Mock(-)
dilution	1	2	3	4	5	6	7	8	9	10	11	12
$1:2^2$				-	-	-	-	-	-	500 $TCID_{50}$/mL		-
$1:2^3$				-	-	-	-	-	-	500 $TCID_{50}$/mL		-
$1:2^4$				-	-	-	+	+	-	500 $TCID_{50}$/mL		-
$1:2^5$				-	-	-	+	+	+	500 $TCID_{50}$/mL		-
$1:2^6$				-	+	-	+	+	+	50 $TCID_{50}$/mL		-
$1:2^7$				+	+	+	+	+	+	5 $TCID_{50}$/mL		-
$1:2^8$				+	+	+	+	+	+	0.5 $TCID_{50}$/mL		-
$1:2^9$				+	+	+	+	+	+	0.05 $TCID_{50}$/mL		-

c

$$nAb\ titer = 2^{\left[D+\left(\frac{a-50}{a-b}\right)\right]}$$

d

sample 1

$$nAb\ titer = 2^{\left[6+\left(\frac{66.\dot{6}-50}{66.\dot{6}-0}\right)\right]} = 2^{(6+0.25)}$$

sample 2

$$nAb\ titer = 2^{\left[3+\left(\frac{100-50}{100-33.\dot{3}}\right)\right]} = 2^{(3+0.75)}$$

e

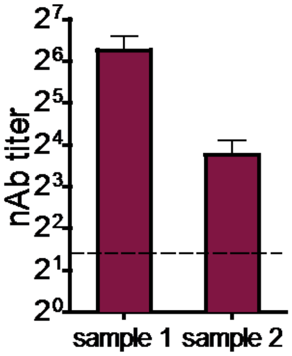

Supplementary Figure 7. Example of HRV serum nAb assay. (a) Raw data; HeLa-H1 cells in 96-well plate after nAb assay described in materials and methods, stained with crystal violet. (b) Experimental plate layout and CPE scores. No cells in columns 1- 3. Cells in columns 4-6 infected with 50 µl of a 1:1 mixture of 500 $TCID_{50}$/ml HRV-14 and two-fold serially diluted serum sample #1. Cells in columns 7-9 infected with 50 µl of a 1:1 mixture of 500 $TCID_{50}$/ml HRV-14 and two-fold serially diluted serum sample #2. Final serum dilution series indicated in leftmost column. Cells in columns 10-11 infected with HRV-14 (without serum) at indicated titers as positive control. Cells in column 12 mock-infected with basal MEM. CPE positive wells denoted by white boxes with + sign; CPE negative wells denoted by blue boxes with – sign. (c) The formula for nAb titer. D exponent of dilution factor to base 2 at dilution immediately above 50% neutralization; a % neutralized at dilution immediately above 50% neutralization; b % neutralized at dilution immediately below 50% neutralization. (d) Calculation of nAb titers in sample 1 and sample 2. (e) Visual presentation of calculated nAb data.

Supplementary Table 1. HRV types and titer in 1-, 3-, 5-, 7-, and 10-valent vaccine. Inactivated-$TCID_{50}$ per 100 µl i.m. dose used in 1-valent, 3-valent, 5-valent, 7-valent, and 10-valent inactivated HRV vaccines depicted in **Figure 1**.

HRV type[1]	1-valent	3-valent	5-valent	7-valent	10-valent
HRV-16	1.7×10^7	3.5×10^6	3.5×10^6	3.5×10^6	3.2×10^6
HRV-36		1.1×10^7	1.1×10^7	1.1×10^7	1.0×10^7
HRV-78		6.3×10^5	6.3×10^5	6.3×10^5	5.6×10^5
HRV-38			1.1×10^5	1.1×10^5	1.0×10^5
HRV-13			6.3×10^5	6.3×10^5	5.6×10^5
HRV-29				3.5×10^4	3.2×10^4
HRV-14				3.5×10^6	3.2×10^6
HRV-76					1.9×10^4
HRV-2					3.2×10^4
HRV-9					5.6×10^5

[1]HRV types chosen to represent eight previously described antigenic groupings of human rhinoviruses, with the addition of HRV-16 and HRV-14 as prototypical types[1]

Supplementary Table 2. HRV types and titers in 10-, and 25-valent vaccines. Inactivated- TCID$_{50}$ per 100 µl i.m. dose used in 10-valent and 25-valent inactivated HRV vaccines depicted in **Figure 3**.

HRV type	10-valent[1]	25-valent[2]	25-valent[3]
HRV-76	1.9×10^{4}	7.7×10^{3}	7.7×10^{3}
HRV-29	1.0×10^{5}	4.1×10^{4}	4.1×10^{4}
HRV-9	2.1×10^{6}	8.6×10^{5}	8.6×10^{5}
HRV-14	3.3×10^{6}	1.3×10^{6}	1.3×10^{6}
HRV-16	1.0×10^{7}	4.3×10^{6}	4.3×10^{6}
HRV-78	1.4×10^{7}	5.7×10^{6}	5.7×10^{6}
HRV-38	1.9×10^{7}	7.7×10^{6}	7.7×10^{6}
HRV-13	2.1×10^{7}		
HRV-2	3.1×10^{7}	2.3×10^{6}	2.3×10^{6}
HRV-36	3.2×10^{7}	1.3×10^{7}	1.3×10^{7}
HRV-32		2.3×10^{3}	**2.3×10^{4}**
HRV-49		2.3×10^{4}	**2.3×10^{5}**
HRV-58		1.2×10^{5}	**2.3×10^{5}**
HRV-55		1.3×10^{5}	**2.3×10^{6}**
HRV-11		1.8×10^{5}	1.8×10^{5}
HRV-41		2.3×10^{5}	**1.3×10^{6}**
HRV-33		3.2×10^{5}	**2.3×10^{6}**
HRV-39		3.2×10^{5}	**2.3×10^{6}**
HRV-50		3.2×10^{5}	**2.3×10^{6}**
HRV-94		3.2×10^{5}	3.2×10^{5}
HRV-1B		4.1×10^{5}	**1.3×10^{6}**
HRV-21		4.1×10^{5}	**2.3×10^{6}**
HRV-51		4.1×10^{5}	4.1×10^{5}
HRV-60		5.1×10^{5}	**1.3×10^{6}**
HRV-28		2.3×10^{6}	2.3×10^{6}
HRV-45		3.3×10^{6}	3.3×10^{6}

[1]HRV types chosen to represent eight previously described antigenic groupings of human rhinoviruses, with the addition of HRV-16 and HRV-14 as prototypical types[1].

[2]Used for prime vaccination. The HRV types added to expand the vaccine were arbitrarily chosen to broadly represent the A species based on phylogeny[2].

[3]Used for boost vaccination. In the interim between the prime and boost vaccination, we obtained higher titer virus stocks of eleven input types (bold font). Higher titers of these eleven were used in the boost vaccination.

Supplementary Table 3. HRV types and titers in 25-, and 50-valent vaccines. Inactivated- $TCID_{50}$ per 1.0 ml i.m. dose used in 25-valent and 50-valent inactivated HRV vaccines depicted in **Figure 4**.

HRV type	25-valent	50-valent[1]
HRV-1B	1.4×10^{7}	7.0×10^{6}
HRV-2	2.4×10^{7}	1.2×10^{7}
HRV-9	8.9×10^{6}	4.4×10^{6}
HRV-11	1.9×10^{6}	1.0×10^{6}
HRV-14	1.4×10^{7}	7.0×10^{6}
HRV-16	4.4×10^{7}	2.2×10^{7}
HRV-21	2.4×10^{7}	1.2×10^{7}
HRV-28	2.4×10^{7}	1.2×10^{7}
HRV-29	4.2×10^{5}	2.1×10^{5}
HRV-32	2.4×10^{5}	1.2×10^{5}
HRV-33	2.4×10^{7}	1.2×10^{7}
HRV-36	1.4×10^{8}	7.0×10^{7}
HRV-38	8.0×10^{8}	4.0×10^{8}
HRV-39	2.4×10^{7}	1.2×10^{7}
HRV-41	1.4×10^{7}	7.0×10^{6}
HRV-45	3.7×10^{7}	1.8×10^{7}
HRV-49	2.4×10^{6}	1.2×10^{6}
HRV-50	2.4×10^{7}	1.2×10^{7}
HRV-51	4.2×10^{6}	2.1×10^{6}
HRV-55	2.4×10^{7}	1.2×10^{7}
HRV-58	2.4×10^{6}	1.2×10^{6}
HRV-60	1.4×10^{7}	7.0×10^{6}
HRV-76	2.4×10^{5}	1.2×10^{5}
HRV-78	5.9×10^{7}	2.9×10^{7}
HRV-94	3.3×10^{6}	1.6×10^{6}
HRV-7		1.2×10^{6}
HRV-10		1.2×10^{7}
HRV-13		4.4×10^{6}
HRV-19		1.4×10^{7}
HRV-24		1.2×10^{7}
HRV-30		1.2×10^{7}
HRV-31		6.7×10^{6}
HRV-34		6.7×10^{6}
HRV-40		2.1×10^{6}
HRV-53		1.2×10^{5}
HRV-54		6.7×10^{7}
HRV-56		1.2×10^{6}
HRV-59		6.7×10^{6}
HRV-64		1.2×10^{6}
HRV-66		4.0×10^{7}
HRV-68		1.6×10^{6}
HRV-75		1.2×10^{7}
HRV-77		2.1×10^{5}
HRV-80		1.0×10^{8}
HRV-81		6.7×10^{6}
HRV-85		4.4×10^{7}
HRV-88		1.6×10^{6}
HRV-89		2.1×10^{6}
HRV-96		4.4×10^{6}
HRV-100		1.2×10^{7}

[1] The HRV types added to expand the vaccine were arbitrarily chosen to broadly represent the A species based on phylogeny[2]

Supplementary Table 4. **Titers of HRV-16, 36 and 78.** Inactivated-$TCID_{50}$ per 100 µl i.m. dose used in non-purified and semi-purified inactivated HRV vaccines depicted in **Supplementary Figure 5**.

HRV type	Non-purified	Semi-Purified
HRV-16	1.7×10^{7}	3.2×10^{6}
HRV-36	3.2×10^{7}	1.1×10^{7}
HRV-78	1.4×10^{7}	5.6×10^{5}

Supplementary References

1. Cooney MK, Fox JP, Kenny GE. Antigenic groupings of 90 rhinovirus serotypes. *Infect Immun* **37**, 642-647 (1982).

2. Waman VP, Kolekar PS, Kale MM, Kulkarni-Kale U. Population structure and evolution of Rhinoviruses. *PLoS One* **9**, e88981 (2014).

3. Kattur Venkatachalam AR, Szyporta M, Kiener TK, Balraj P, Kwang J. Concentration and purification of enterovirus 71 using a weak anion-exchange monolithic column. *Virol J* **11**, 99 (2014).

4. Weiss VU, *et al.* Capillary electrophoresis, gas-phase electrophoretic mobility molecular analysis, and electron microscopy: effective tools for quality assessment and basic rhinovirus research. *Methods Mol Biol* **1221**, 101-128 (2015)
